# Neurocysticercosis: HP10 Antigen Detection Is Useful for the Follow-up of the Severe Patients

**DOI:** 10.1371/journal.pntd.0002096

**Published:** 2013-03-07

**Authors:** Agnès Fleury, Esperanza Garcia, Marisela Hernández, Roger Carrillo, Tzipe Govezensky, Gladis Fragoso, Edda Sciutto, Leslie J. S. Harrison, R. Michael Evans Parkhouse

**Affiliations:** 1 Unidad Periférica, Instituto de Investigaciones Biomédicas, UNAM / Instituto Nacional de Neurología y Neurocirugía, Colonia la Fama, Delegación Tlalpan, México DF, México; 2 Departamento de Inmunología, Instituto Nacional de Neurología y Neurocirugía, México DF, México; 3 Departamento de Inmunología, Instituto de investigaciones Biomédicas, UNAM, México DF, México; 4 Departamento de Neuroradiología, Instituto Nacional de Neurología y Neurocirugía, México DF, México; 5 Apoyo a Estadística, Instituto de Investigaciones Biomédicas, UNAM, México DF, México; 6 Division of Veterinary Clinical Sciences, Incorporating Centre for Tropical Veterinary Medicine Royal (Dick) School of Veterinary Studies, University of Edinburgh, Easter Bush Veterinary Centre, Scotland, United Kingdom; 7 Instituto Gulbenkian de Ciencia, Oeiras, Portugal; Institute of Tropical Medicine of Antwerp, Belgium

## Abstract

**Background:**

The most severe clinical form of neurocysticercosis (NC) occurs when cysticerci are located in the subarachnoid space at the base of the brain (SaB). The diagnosis, monitoring and treatment of NC-SaB, constitutes a severe clinical challenge. Herein we evaluate the potential of the HP10 antigen detection enzyme-linked immunosorbent assay (HP10 Ag-ELISA) in the long term follow-up of NC-SaB cases. Assay performance was compared with that of Magnetic Resonance Imaging (MRI). In addition, the robustness of the HP10 Ag-ELISA was evaluated independently at two different institutions.

**Methodology/Principal Findings:**

A double-blind prospective cohort trial was conducted involving 38 NC-SaB cases and a total of 108 paired serum and cerebrospinal fluid (CSF) samples taken at intervals of 4 to 8 months for up to 43 months. At each medical visit, results of sera and CSF HP10 Ag-ELISA and MRI obtained at last visit were compared and their accuracy was evaluated retrospectively, considering radiological evolution between appointments. In the long-term follow-up study, HP10 Ag-ELISA had a better agreement than MRI with retrospective radiological evaluation. High reproducibility of HP10 Ag-ELISA between laboratories was also demonstrated.

**Conclusions:**

Results reported in this study establish for the first time the usefulness of the comparatively low cost HP10 Ag-ELISA for long term follow-up of NC-SaB patients.

## Author Summary

Neurocysticercosis is one of the most frequent parasitic diseases affecting the human central nervous system. The most severe clinical forms occur when parasites are located in the subarachnoid space at the base of the brain. In these instances, cysticidal drug efficacy is reduced and neuroimaging studies are less reliable as diagnostic tools. Previous works highlighted the value of antigen detection by ELISA test to detect viable parasites in these locations. In this prospective study, we evaluate its utility in patient follow-up, comparing its performance with magnetic resonance imaging results. Results from both procedures were also compared retrospectively at each medical appointment considering radiological evolution since last evaluation. Thirty-eight patients were included, with a total of 108 samples collected over 43 months. We demonstrated that antigen detection in these patients is an accurate tool in determining the efficacy of cysticidal treatment. This result is of great potential, considering the difficulty for the patients in endemic countries to access imaging studies and the much lower cost of the assay with respect to magnetic resonance imaging.

## Introduction

Neurocysticercosis (NC) is one of the most frequent parasitic diseases affecting the human central nervous system [1]. It is transmitted by the ingestion of *Taenia solium* eggs mainly through the consumption of contaminated vegetables, and is still prevalent in most countries of Asia, Africa and Latin America, including México [2]. Additionally, its prevalence is rising in the United States and some European countries due to increasing immigration [3], [4].

NC severity critically depends on the location of the parasite. The disease is mostly benign when cysticerci are located in the cerebral parenchyma, neuroimaging techniques accurately indicating the number, localization, viability of the parasites and the intensity of the inflammatory reaction [5], [6]. In contrast, when parasites are located in the basal subarachnoid space (NC-SaB), clinical presentation is generally severe, cysticidal drugs are less effective and neuroradiological studies are less precise, diagnosis relying mostly on indirect clues such as the enlargement of the basal cisternae [7], [8].Furthermore, neuroradiological studies represent the most expensive healthcare-related costs [9], and are only available at major urban centres whereas the principal population at risk is mostly rural.

Detection of the secreted metacestode antigens, particularly HP10 [10] is becoming an increasingly accepted test for diagnosing severe NC [11]–[19]. Previous studies have demonstrated the high specificity and sensitivity of HP10 antigen ELISA assay to detect NC-SaB [14]–[18], and its similar accuracy either when sera or CSF samples are employed [12], [15], [18].

In this first prospective long-term study focusing on NC-SaB, we evaluated the assay reproducibility and accuracy, comparing the MRI and HP10 results and considering the radiological evolution of the patients retrospectively at each medical appointment.

## Methods

### Study cases, MRI examination and sampling

This prospective longitudinal study was performed in a total of thirty-eight NC-SaB patients who attended to the Instituto Nacional de Neurología y Neurocirugía (INNN), Mexico City. The patients included were selected consecutively between August 2008 and March 2010. Initially, fifty patients were included, but 8 were lost in the follow-up as they did not come to the second medical appointment and, in 4 cases, paired CSF and serum HP10 determinations were not made as their increased intracranial pressure precluded the taking of CSF samples. Diagnosis was based on clinical manifestations (presence of focal deficit, affection of cranial nerves and intracranial hypertension), imaging studies (MRI with images compatible with the presence of cysticerci, ie. mainly, enlargement or deformation of a basal cistern or visualization of cystic vesicles), and HP10 positive in CSF. Patients were followed-up during 6 to 43 months, resulting in a total of 108 individual clinical, radiological, and serological evaluations. Each of the 38 cases had received between 1 to 7 cycles of cysticidal drug treatment before being included in the trial. In most cases albendazole (ABZ) was used at a dose of 30 mg/kg/day during 8 consecutive days. Few patients received praziquantel (PZQ, 50 mg/kg/day during 10 days) or a combination of ABZ plus PZQ. Cysticidal drugs were always accompanied with prednisone (1 mg/kg/day) or dexamethasone (0.4 mg/kg/day), followed by individual decreasing prednisone doses, depending on the clinical and inflammatory status.

MRI results (gadolinium-enhanced T1 and T2- weighted and FLAIR, (Fluid Attenuation Inversion Recovery), MRI sequences) were recorded, and paired CSF/serum samples were tested for HP10 Ag-ELISA. Follow-up was carried out at least once in all cases, at 4–8 month intervals, with further combined HP10 Ag-ELISA and MRI studies. The time between CSF/serum sampling for HP10 Ag-ELISA and the associated MRI examination was ≤1 month. During this time, no cestocidal treatments were administered.

At each medical visit, changes in MRI studies since last appointment were assessed and, based on these criteria, the accuracy of MRI and HP10 results at last evaluation was established. We considered that patient were free of SaB vesicular parasites at last evaluation if we did not see any changes in the suspect images on MRI (absence of disappearance or diminution in case of specific treatment or enlargement if no cestocidal treatment was administered). On the other hand, we concluded that patients present SaB vesicular parasites at their last evaluation if we observed a reduction of the suspected image after treatment or its enlargement if treatment had not been administered.

All MRI studies were double-blind interpreted by a certified neuroradiologist with extensive experience in NC diagnosis. MRI was considered positive if a vesicular cyst located in the subarachnoid basal cisterns or a deformation of a cistern compatible with the presence of a vesicular cyst were observed.

### Ethics statement

The study was approved by and carried out under the guidelines of the Ethical Committee of the Instituto Nacional de Neurología y Neurocirugía, D.F., México. All patients provided written informed consent for the collection of samples and subsequent analysis.

### HP10 Ag-ELISA

HP10 Ag-ELISA were blindly performed by two Mexican laboratories (Instituto de Investigaciones Biomedicas, UNAM (IIB), and INNN), both in Mexico City, Mexico. Afterwards, sample codes were revealed and results were analyzed.

HP10 antigen was detected by Ag-ELISA as described previously [10]. All samples were run in duplicate by two experimented technicians. Briefly, plates (Nunc, Rochester, New York, USA) were coated with monoclonal antibody (MoAb) HP10 (100 µl at 10 µg/ml in 0.07 M NaCl buffered with 0.1 M borate, pH 8.2) and left overnight at 4°C, washed four times with 200 µl/well of wash solution (0.9% w/v NaCl containing 0.05%v/v Tween 20) and then blocked using 200 µl of phosphate-buffered saline containing bovine serum albumin (Roche, México) (1.0% w/v and 0.05% v/v Tween 20) and left by 60 min at room temperature before being washed in a similar way. Undiluted CSF or serum samples (100 µl/well) were added and incubated 30 min at 37°C. Bound HP10 parasite antigen was detected using biotinylated MoAb HP10 (2 µg/ml in diluent, for 60 min at 37°C), horseradish peroxidase-conjugated streptavidin (Zymed, San Francisco, California, USA) (1∶4000 in diluent, 30 min at 37°C) and tetramethylbenzidine (Zymed, San Francisco, California, USA) as substrate. Color reaction was allowed to proceed for 30 min at 4°C in the dark and was stopped by adding 100 µl 0.2 M H_2_SO_4_ (Baker, Estado de Mexico, Mexico). Optical densities (OD; 450 nm) were determined in an ELISA processor (at INNN: Bio-Rad Microplate Reader Benchmark, Hercules, California, USA; at IIBM: Opsys MR Dynex Technology, Chantilly, Virginia, USA).

A sample was considered positive if the mean OD at 450 nm value was higher than the cut off value, which was calculated based on the mean of the OD plus 2 SD of CSF and sera from non-NC controls.

### Statistical analysis

A database was built using Excel 7.0 (Microsoft) software. MRI findings and CSF/serum HP10 levels were recorded. Statistical analysis was made using SPSS 10.0 (Microsoft), Epidat 3.0 and SAS 9.0 softwares. Parametric statistics (mean and SD) were calculated. Kappa coefficient (*k*) and 95% confidence intervals were calculated to evaluate the qualitative agreement between HP10 results in CSF and sera and between institutions. This coefficient varied between −1 and +1. The closer the value is to1, the stronger the agreement [20]. Generalized Estimating Equations (GEE) analysis [21] was performed in order to confirm agreement taking into account repeated observations and variability of period of time between appointments. Results confirm that these two factors did not modify the agreement between tests.

## Results

### Summary of case descriptions

The main features of the 38 patients included in the study are summarized in table 1. Most patients had vesicular cysticerci located in the SaB with inflammatory CSF, in spite of the previous cysticidal treatment.

**Table 1:** Main features of the 38 patients included in the study.

Sex (Male/Female)	24/14
Percent of patients with vesicular cysticerci in SaB	63% (24/38)
Age	29–67 (46.3±10.8)*
Number of cysticidal cycles received before inclusion	0–8 (2.4±2.0)*
CSF cellularity at inclusion	1–343 (58.9±67.3)*
Number of samples per patient	2–5 (2.1±1.0)*
Months of follow-up	6–39 (27.2±9.1)*

* Range (mean±SD).

### Reproducibility between institutions in assay performance

Kappa analysis indicated a good to very good level of agreement between positive/negative allocations comparing results obtained in both institutions (CSF Kappa: 0.86 [0.75–0.97, *P*<0.0001] and serum Kappa: 0.76 [0.64–0.89, *P*<0.0001]) [15].

### HP10-ELISA performed equally well in CSF than in serum

A good level of agreement^15^ between positive/negative allocations for paired CSF and serum samples (CSF/serum IIBM Kappa: 0.63 [0.48–0.79, *P*<0.0001] and CSF/serum INNN Kappa: 0.64 [0.49–0.79, *P*<0.0001]) was found.

### Comparison of MRI and CSF HP10 Ag-ELISA results

There was complete agreement between radiological and CSF HP10 Ag-ELISA results in 18 out of the 38 cases (50 samples).

As shown in table 2, as judged by negative MRI and CSF HP10 Ag-ELISA results, the infection resolved only in three of these cases. In the other 15 cases, cysticerci persisted and so additional ABZ cycles were indicated.

**Table 2:** Main features of the 18 patients with complete agreement between CSF HP10 Ag-ELISA and MRI evaluation.

Number of evaluations per patient*	Number of patients (total number of evaluations)	Results of last evaluations
2	9 (18)	9 positives†
3	5 (15)	3 positives/2 negatives
4	3 (12)	2 positives/1 negative
5	1 (5)	1 positive
**Total:**	**18 (50)**	**15 positives/3 negatives**

* Evaluations refer to the paired realization in each patient of MRI and of HP10 determination in CSF and serum. The results of HP10 considered in this table are those obtained at IIB in CSF.

† Positive: persistence of vesicular cysticerci in SaB (using MRI or HP10 antigen levels)/Negative: No detectable vesicular cysticerci in SaB (using MRI or HP10 antigen levels).

Of the remaining 20 patients without complete agreement between MRI and CSF HP10 Ag-ELISA assays in all samples, discordant results were found in 32 of the 58 samples (55.2%).

### Comparison of MRI and sera HP10 Ag-ELISA results

As shown in table 3, a complete agreement between HP10 Ag-ELISA in sera and MRI evaluation was obtained in 14 patients, corresponding to 38 paired samples. In the remaining 24 patients, disagreement was observed in some of the samples (43 of 70, 61%).

**Table 3:** Main features of the 14 patients with complete agreement between sera HP10 Ag-ELISA and MRI evaluations.

Number of evaluations per patient*	Number of patients (total number of evaluations)	Results of last evaluation
2	8 (16)	7 positives/1 negative†
3	3 (9)	3 positives
4	2 (8)	2 positives
5	1 (5)	1 positive
**Total:**	**14 (38)**	**13 positives/1 negative**

* Evaluations refer to the paired realization in each patient of MRI and of HP10 determinations in sera obtained at IIB.

† Positive: persistence of vesicular cysticerci in SaB (using MRI or HP10 levels)/Negative: No detectable vesicular cysticerci in SaB (using MRI or HP10 levels).

### Comparison of MRI and retrospective radiological evolution

As shown in table 4, agreement between MRI results and retrospective evaluation taking in count radiological evolution between appointment was moderate: Kappa: 0.45 (0.26–0.63). In 24 samples, divergent results occurred: 15 of them with positive results in MRI and negative results in retrospective evaluation, and the reverse in 9 of them.

**Table 4:** Agreement analysis between MRI results and retrospective evaluation.

		Retrospective evaluation		Total
		Presence of parasites	Absence of parasites	
MRI	Presence of parasites	66	15	81
	Absence of parasites	9	18	27
Total		75	33	108

### Comparison of CSF HP10 Ag-ELISA and retrospective radiological evolution

As shown in table 5, agreement between CSF HP10 Ag-ELISA results and retrospective evaluation taking in count clinical and radiological evolution was very good: Kappa: 0.82 (0.70–0.94). Differences between the evaluations occurred in only 8 samples, with positive results in HP10 Ag-ELISA/negative results in retrospective clinical/radiological evaluations in 6 and the reverse in two.

**Table 5:** Agreement analysis between CSF HP10 results and retrospective evaluation.

		Retrospective evaluation		Total
		Presence of parasites	Absence of parasites	
HP10 CSF	Positive	73	6	79
	Negative	2	27	29
Total		75	33	108

### Comparison of sera HP10 Ag-ELISA and retrospective radiological evolution

As shown in table 6, the agreement between sera HP10 Ag-ELISA results and retrospective evaluation taking in count clinical and radiological evaluation was moderate: Kappa: 0.56 (0.39–0.73). Differences between the evaluations occurred in 21 samples, with positive results in sera HP10 Ag-ELISA/negative results in retrospective clinical/radiological evaluations in 8 and the reverse in 13.

**Table 6:** Agreement analysis between sera HP10 results and retrospective evaluation.

		Retrospective evaluation		Total
		Presence of parasites	Presence of parasites	
HP10 sera	Positive	62	8	70
	Negative	13	25	38
Total		75	33	108

## Discussion

Diagnosis of NC- SaB is still a challenge. In previous studies, it was demonstrated that the levels of secreted cysticercal HP10 antigen in CSF and serum is an accurate method to diagnose vesicular cysticerci located in the ventricles or SaB [11]–[15]. HP10 Ag-ELISA was originally designed to diagnose *Taenia saginata* cysticercosis [10], but since HP10 antigen is shared by other cestodes such as *T. solium*, it is also useful for NC diagnosis [11]–[15].

In this prospective longitudinal study, we evaluated the usefulness of the HP10 Ag-ELISA assay in the follow-up of patients with NC-SaB, and compared its predictive capacity with MRI, the best tool currently available for the diagnosis and follow-up of these patients. Both tests were compared with a retrospective diagnosis based on radiological evolution between medical appointments.

The results obtained herein demonstrate the usefulness of HP10 Ag-ELISA for the follow-up of severe NC-SaB patients. Of the 108 paired MRI/HP10 Ag-ELISA evaluations, agreement between MRI and CSF HP10 Ag-ELISA was found in 76 (70.4%) and between MRI and sera HP10 in 65 (60%). Retrospective analysis evaluating at each medical appointment the radiological evolution between visits shows that this lack of agreement appears to be principally due to a misinterpreted MRI rather than to serum and/or CSF HP10 Ag-ELISA assay. In addition, HP10 Ag-ELISA proved to be a highly reproducible method, as high Kappa coefficients of 0.76 and 0.86 in sera and CSF, respectively, were obtained when comparing results obtained in two different Mexican institutions.

Another result meriting comment is the confirmation that determination of HP10 in sera is of interest, giving quite similar agreement than MRI with retrospective diagnosis. Agreement between CSF HP10 determination and retrospective evaluation was higher, but considering the invasive nature of the lumbar puncture, procedure necessary to collect CSF, the use of sera instead of CSF could be recommended, particularly if there is no clinical emergency, and also for monitoring of SaB neurocysticercosis patients from rural communities where imaging facilities are not available.

Considering these results, it is possible to propose HP10 determination in order to reduce the number of MRI required in the follow-up of such patients. Generally, MRIs are realized each 6 months to evaluate response to treatment, and frequently 4 to more than 8 studies are made by patient during their illness and treatment. The results herein presented show that it will be possible to reduce the number of studies to at least a half without taking a risk of misdiagnosis. We recommend that after diagnosis of NC-SaB by MRI (diagnosis must be made by MRI as it will permit the evaluation of the extent of the disease), the follow-up of the patients could be made by one MRI and one HP10 evaluation each year until parasites disappears. Election of serum or CSF HP10 evaluation, as said before, will depend on the gravity of the patients and the possibility to make lumbar puncture without risk.

Importantly, the results obtained in this study point to the need for improving MRI for NC diagnosis. As shown here, when cysticerci are located in the SaB, diagnostic radiology techniques are imprecise. This can be explained by the fact that parasites emit a signal of similar intensity than the CSF itself. In addition, in most cases the image is not enhanced by administration of intravenous contrast, and finally, metacestodes commonly lack the distinguishing scolex that allows their identification [8]. In this respect, new MRI technologies based on a fast imaging employing steady-state acquisition (FIESTA) has shown to have a good capacity to diagnose intraventricular cysts because of their high spatial resolution and signal-to-noise rate [22]. Nevertheless, despite the promising results reported, these procedures are not yet standardized for NC diagnosis. It is also interesting to note that FLAIR sequences, permitting better visualization of the scolex and the cyst wall, did not resolve all the cases in our studies [5], [23].

The need for an economic and reliable diagnosis of cysticercosis is now of urgent concern in rural cysticercosis endemic communities, particularly as recent plans for the control of neglected tropical diseases, such as schistosomiasis, involve the mass and indiscriminate dosing of entire African populations with antihelmintic drugs, including praziquantel [24]. Such treatment, of population co-infected with *T. solium* neurocysticercosis, would be predicted to increase the inflammation around the cyst, thereby provoking severe side effects [25]–[27]. Thus a prior screening for cysticercosis/neurocysticercosis, ideally including antibody and antigen detection, might be advisable.

Neuroradiological studies required for diagnosis and patient follow-up currently represent the main cost of NC management in particular in severe NC-SaB, which usually requires multiple cysticidal cycles of treatment [9]. Thus, the alternative use of a serological non-invasive, non-expensive, highly accurate assay could have a real economic impact. To illustrate this point, is worthy to mention that an MRI in Mexico costs approximately 400 dollars, a cost dramatically contrasting with approximately 1–10 dollars for a typical commercial Ag-ELISA test. HP10 ELISA could have an undeniably positive economic impact for the patient and for the health institutions as well. This study offers arguments to strongly recommend its routine use for the follow-up of these severely affected patients.

Despite the HP10 Ag-ELISA accuracy, HP10 false positive results would lead to unnecessary treatment of these patients (being corticoid administration the main problem).Thus, it is urgent to investigate the factors underlying false positive results. False negative HP10 results were observed only in 2 of the 108 evaluations (1.8%) in CSF and in 13 (12%) in serum, posing a very low risk of failing to discriminate a NC-SaB patient still requiring treatment. To avoid the risk of incomplete drug treatment, we suggest repeating serum HP10 Ag-ELISA test in patients presenting NC-SaB with HP10-negative results after treatment.

It is important to note that these results must be confirmed. Limitations of this study are mainly due to: 1) the small number of patients that leads to large confidence intervals and uncertainty. However, it must be stated that these types of patients (NC-SaB) are infrequent and that this study is the first one with such large follow-up; 2) the retrospective diagnosis can be criticized as it is known that evolution of parasites is very slow. It is possible that in some cases, for example, radiological picture of the patients did not change between 2 appointments mistakenly making believe that there were no parasites although there were present. We are conscious of this fact and we hope that the standardization of new MRI techniques will permit to give new tools to evaluate these tests.

In conclusion, this study establishes the usefulness and economic advantage of the HP10 Ag-ELISA applied on CSF and serum samples for the follow-up of patients with NC-SaB, the most severe form of the disease. Hopefully, these results will lead to the rapid commercialization of a HP10 antigen diagnostic kit to favor its employment worldwide.
